# Supplementary material for: A Likelihood Approach for Real-Time Calibration of Stochastic Compartmental Epidemic Models
Source: PLoS Comput Biol. 2017 Jan 17;13(1):e1005257. doi: 10.1371/journal.pcbi.1005257 (PMC5240920; doi:10.1371/journal.pcbi.1005257)
Supplement: S1 File — (TAR.GZ) [file pcbi.1005257.s014.tar.gz › HSPH_Online-SI-Revision/MSS10/n1-severe/n1-severe_NEW_table8.pdf]

1	R0	{1.51081, 13.7078, 1.}	{1.46631, 3.94819, 0.04}	{{1.44762, 3.83841, 0.}, {1.47608, 4.06661, 0.}}	{3.10552, 71.1975, 102.}	inf	54.	10.4179	{2.66949, 16.6271}	80.7076	0.796822	newly	28.	14.71	{3., 27.}	50.9643	0.391149	Reff	0.70857	0.751593	{0.735912, 0.76729}	6.23474	ar	656.	510.15	{482., 541.}	22.2332
2	R0	{1.73182, 11.3873, 3.}	{1.63628, 3.48956, 0.05}	{{1.53501, 3.37285, 0.}, {1.65456, 4.02511, 0.}}	{5.51689, 69.3557, 98.3333}	inf	39.	10.7322	{3.39691, 18.2151}	72.4815	0.629156	newly	19.	16.6	{5., 28.}	38.5263	0.239692	Reff	0.680605	0.689265	{0.654841, 0.707407}	2.6687	ar	653.	602.33	{576., 631.}	7.82695
3	R0	{1.50449, 10.3474, 0.1}	{1.49591, 3.88189, 0.08}	{{1.47488, 3.83841, 0.}, {1.53501, 4.02511, 0.}}	{2.14298, 62.4844, 170.}	inf	38.	11.8316	{4.66919, 18.7193}	68.8641	0.571568	newly	22.	15.96	{6., 27.}	38.9091	0.25271	Reff	0.664985	0.700353	{0.679132, 0.725105}	5.32049	ar	651.	555.96	{529., 587.}	14.5991
4	R0	{1.63493, 9.1955, 0.1}	{1.66491, 8.77052, 0.44}	{{1.58886, 7.29751, 0.}, {1.71608, 9.75208, 1.}}	{4.02301, 11.7639, 458.}	inf	17.	23.8501	{13.9271, 33.2808}	47.9803	0.16412	newly	9.	16.06	{10., 23.}	81.7778	0.246939	Reff	0.614734	0.614259	{0.593472, 0.63342}	2.2325	ar	663.	664.2	{638., 692.}	2.62745
5	R0	{1.53822, 8.05553, 0.1}	{1.62271, 10.8957, 2.26}	{{1.56097, 8.77207, 2.}, {1.79724, 16.8903, 3.}}	{5.49249, 36.4203, 2162.}	inf	26.	56.6333	{33.997, 103.029}	118.605	0.297677	newly	17.	30.84	{22., 39.}	81.6471	0.248141	Reff	0.642976	0.625942	{0.609303, 0.639858}	2.8794	ar	621.	682.48	{627., 764.}	10.1031
6	R0	{1.69374, 17.0793, 0.1}	{1.75588, 19.2972, 1.}	{{1.75588, 19.2972, 1.}, {1.75588, 19.2972, 1.}}	{3.66892, 12.9859, 900.}	inf	76.	54.3962	{48.3655, 61.5118}	28.4261	0.147427	newly	35.	19.02	{13., 25.}	45.6571	0.276505	Reff	0.753714	0.819301	{0.805102, 0.829753}	8.70184	ar	662.	651.45	{604., 693.}	4.41239
7	R0	{1.42179, 11.8031, 2.}	{1.54207, 13.1766, 1.98}	{{1.54363, 13.2419, 2.}, {1.54363, 13.2419, 2.}}	{8.50742, 12.4988, 1.}	inf	20.	43.7196	{38.3906, 50.6398}	120.471	0.349745	newly	9.	22.02	{17., 28.}	146.444	0.390388	Reff	0.720848	0.745189	{0.735158, 0.753652}	3.37672	ar	517.	608.28	{567., 647.}	17.8182
8	R0	{1.46683, 6.95875, 0.1}	{1.67462, 7.30709, 1.02}	{{1.67405, 7.28757, 1.}, {1.67405, 7.28757, 1.}}	{14.166, 5.00578, 920.}	inf	0.1	11.5262	{7.70557, 14.8122}	11426.2	2.04497	newly	0.1	9.07	{5., 13.}	8970.	1.9288	Reff	0.50899	0.561794	{0.556261, 0.567818}	10.3743	ar	653.	677.72	{666., 692.}	3.7856
9	R0	{1.34525, 11.505, 1.}	{1.2559, 2.79591, 0.08}	{{1.24751, 2.5364, 0.}, {1.25474, 3.26445, 0.}}	{7.01976, 75.6983, 102.}	inf	36.	8.27682	{1.99681, 15.7543}	77.3414	0.755583	newly	22.	15.08	{5., 28.}	49.2727	0.338791	Reff	0.768138	0.751859	{0.738081, 0.755641}	2.86573	ar	532.	425.14	{398., 470.}	20.3647
10	R0	{1.77958, 14.372, 0.1}	{1.66055, 15.2961, 2.}	{{1.66055, 15.2961, 2.}, {1.66055, 15.2961, 2.}}	{6.6888, 6.43017, 1900.}	inf	31.	58.2544	{52.3315, 65.4622}	87.9173	0.272541	newly	16.	24.08	{19., 29.}	50.75	0.172452	Reff	0.619294	0.532613	{0.52054, 0.54178}	13.9967	ar	671.	732.18	{713., 755.}	9.11773
11	R0	{1.60334, 19.9548, 1.}	{1.42136, 17.2398, 3.}	{{1.42136, 17.2398, 3.}, {1.42136, 17.2398, 3.}}	{11.3502, 13.6058, 200.}	inf	70.	55.1283	{48.7805, 60.7496}	21.2453	0.105384	newly	28.	21.2	{16., 27.}	24.7857	0.131017	Reff	0.80167	0.731817	{0.723609, 0.740319}	8.71343	ar	617.	584.01	{551., 616.}	5.87196
12	R0	{1.68623, 7.58585, 0.1}	{1.65517, 3.37891, 0.01}	{{1.65456, 3.37285, 0.}, {1.65456, 3.37285, 0.}}	{1.8769, 55.4578, 108.}	inf	7.	4.01662	{1.71256, 6.72369}	46.4066	0.316439	newly	7.	5.83	{2., 10.}	44.4286	0.258436	Reff	0.612101	0.605764	{0.600783, 0.609354}	1.07424	ar	640.	639.98	{633., 649.}	0.93125
13	R0	{1.82689, 18.0022, 1.}	{1.56067, 3.90675, 0.06}	{{1.53501, 3.37285, 0.}, {1.65456, 4.02511, 0.}}	{14.5725, 78.2985, 98.}	inf	56.	8.42416	{2.89855, 13.6627}	84.9569	0.889677	newly	15.	11.36	{3., 20.}	44.9333	0.284492	Reff	0.668642	0.645377	{0.627923, 0.683782}	4.381	ar	673.	601.92	{583., 618.}	10.5706
14	R0	{1.75911, 9.16883, 0.1}	{1.5996, 7.5474, 1.08}	{{1.58886, 7.29751, 1.}, {1.58886, 7.29751, 1.}}	{9.20768, 20.5528, 980.}	inf	25.	19.6432	{12.4188, 26.4305}	25.8138	0.141588	newly	15.	14.99	{9., 20.}	24.7333	0.113322	Reff	0.656148	0.605103	{0.593281, 0.615336}	7.79843	ar	661.	649.1	{623., 674.}	2.86838
15	R0	{1.46168, 11.3859, 2.}	{1.51397, 3.99991, 0.03}	{{1.47488, 3.83841, 0.}, {1.53501, 4.02511, 0.}}	{3.69286, 64.8696, 99.5}	inf	9.	5.16775	{1.92466, 8.84956}	46.6765	0.328861	newly	3.	7.06	{2., 12.}	152.	0.376167	Reff	0.654833	0.684054	{0.662961, 0.69809}	4.48765	ar	556.	559.05	{545., 576.}	1.73561
16	R0	{1.53577, 11.7581, 1.}	{1.53426, 8.13586, 1.54}	{{1.44485, 6.00596, 0.}, {1.56097, 8.77207, 2.}}	{2.62522, 30.8063, 100.}	inf	31.	14.9701	{5.11236, 21.9544}	51.7092	0.36445	newly	13.	10.88	{5., 16.}	30.4615	0.17007	Reff	0.664988	0.688712	{0.659885, 0.703952}	3.88482	ar	625.	581.09	{549., 616.}	7.2048
17	R0	{1.70698, 9.72924, 0.1}	{1.71306, 8.74962, 1.02}	{{1.71337, 8.80249, 1.}, {1.71337, 8.80249, 1.}}	{0.377955, 10.0688, 920.}	inf	8.	31.3173	{25.6357, 37.9619}	293.324	0.589468	newly	4.	20.66	{15., 27.}	417.5	0.700947	Reff	0.571838	0.533921	{0.522125, 0.543742}	6.65283	ar	675.	718.15	{699., 741.}	6.45185
18	R0	{1.5973, 14.931, 0.1}	{1.27002, 3.43818, 0.03}	{{1.22544, 2.5364, 0.}, {1.28596, 3.98513, 0.}}	{20.4896, 76.9729, 124.}	inf	59.	13.5058	{4.42512, 21.221}	77.1087	0.707948	newly	28.	23.74	{8., 40.}	39.	0.22151	Reff	0.967964	0.827368	{0.800182, 0.845298}	14.5249	ar	632.	393.37	{345., 449.}	37.7579
19	R0	{1.87468, 17.004, 1.}	{1.48554, 10.2427, 1.99}	{{1.48482, 10.2395, 2.}, {1.48482, 10.2395, 2.}}	{20.7579, 39.7632, 99.}	inf	48.	20.3722	{15.5358, 24.6761}	57.5579	0.386188	newly	12.	12.04	{8., 16.}	19.1667	0.090654	Reff	0.684258	0.583276	{0.57653, 0.589867}	14.7579	ar	692.	631.05	{613., 649.}	8.8078
20	R0	{1.84715, 12.531, 0.1}	{1.52414, 9.52864, 1.}	{{1.52414, 9.52864, 1.}, {1.52414, 9.52864, 1.}}	{17.4871, 23.9595, 900.}	inf	9.	17.4461	{13.3042, 21.2632}	93.8453	0.280293	newly	3.	11.	{7., 15.}	266.667	0.547394	Reff	0.676057	0.544961	{0.539071, 0.551241}	19.3912	ar	661.	661.68	{647., 677.}	1.35552
21	R0	{1.75938, 13.5959, 2.}	{1.47817, 3.92067, 0.03}	{{1.44762, 3.83841, 0.}, {1.53501, 4.06661, 0.}}	{15.9836, 71.1629, 98.5}	inf	38.	8.2783	{2.653, 13.783}	78.215	0.736816	newly	12.	12.11	{4., 20.}	47.5833	0.24189	Reff	0.777646	0.697279	{0.680011, 0.725579}	10.3346	ar	620.	545.51	{525., 571.}	12.0145
22	R0	{1.63456, 10.7469, 0.1}	{1.65517, 3.37891, 0.01}	{{1.65456, 3.37285, 0.}, {1.65456, 3.37285, 0.}}	{1.26107, 68.5593, 108.}	inf	11.	4.28101	{1.36599, 7.51999}	61.0817	0.474443	newly	7.	6.03	{2., 11.}	43.	0.26674	Reff	0.568827	0.587122	{0.58154, 0.591727}	3.21633	ar	654.	650.92	{643., 661.}	1.02141
23	R0	{1.62702, 19.1392, 1.}	{1.4786, 3.96748, 0.02}	{{1.44762, 3.83841, 0.}, {1.53501, 4.06661, 0.}}	{9.12203, 79.2704, 100.}	inf	42.	8.17583	{2.03377, 13.8071}	80.5337	0.800228	newly	16.	11.47	{3., 19.}	43.4375	0.298815	Reff	0.715889	0.700589	{0.684663, 0.72238}	2.62022	ar	600.	542.88	{522., 565.}	9.56333
24	R0	{1.70466, 12.1202, 2.}	{1.64882, 3.38475, 0.01}	{{1.65456, 3.37285, 0.}, {1.65456, 3.37285, 0.}}	{3.27545, 72.0735, 99.5}	inf	15.	4.6234	{2.00528, 7.89607}	69.1773	0.570631	newly	6.	7.03	{2., 13.}	57.1667	0.244923	Reff	0.685273	0.679934	{0.674579, 0.686462}	0.866329	ar	613.	596.63	{585., 614.}	3.21207
25	R0	{1.61735, 11.3169, 0.1}	{1.71275, 4.85381, 1.14}	{{1.65456, 3.37285, 0.}, {1.80055, 6.15907, 2.}}	{5.89877, 57.11, 1094.}	inf	17.	4.13314	{1.54439, 7.06775}	75.6874	0.675416	newly	11.	4.59	{1., 8.}	58.8182	0.479105	Reff	0.533726	0.587241	{0.567602, 0.618573}	10.0267	ar	681.	661.73	{655., 668.}	2.85609
26	R0	{1.56977, 12.3035, 2.}	{1.4423, 5.38251, 0.24}	{{1.40191, 4.00571, 0.}, {1.47488, 6.00596, 1.}}	{8.12026, 56.2522, 88.}	inf	23.	8.03866	{3.1595, 13.5244}	65.0493	0.515023	newly	15.	8.77	{3., 16.}	47.	0.332566	Reff	0.758199	0.718206	{0.699988, 0.733504}	5.28546	ar	594.	520.33	{500., 549.}	12.4024
27	R0	{1.46317, 5.8298, 0.1}	{1.53354, 4.00369, 0.}	{{1.53501, 4.02511, 0.}, {1.53501, 4.02511, 0.}}	{4.83043, 31.3238, 100.}	inf	0.1	3.0848	{1.48671, 5.02956}	2984.8	1.44243	newly	0.1	4.4	{1., 8.}	4302.	1.53441	Reff	0.605752	0.630152	{0.625231, 0.633336}	4.06852	ar	586.	594.45	{588., 603.}	1.44198
28	R0	{1.70002, 14.9806, 1.}	{1.58389, 10.8293, 3.}	{{1.58389, 10.8293, 3.}, {1.58389, 10.8293, 3.}}	{6.83125, 27.7113, 200.}	inf	50.	24.161	{17.7354, 30.0195}	51.678	0.32657	newly	31.	13.99	{8., 19.}	54.871	0.366333	Reff	0.683408	0.677649	{0.668362, 0.6875}	1.2115	ar	652.	612.46	{586., 643.}	6.14724
29	R0	{1.52729, 16.1014, 3.}	{1.42008, 7.22501, 2.7}	{{1.41695, 6.00596, 0.}, {1.41695, 7.40677, 3.}}	{7.01952, 55.128, 10.}	inf	25.	12.747	{6.59697, 18.1587}	49.0121	0.331795	newly	10.	10.69	{5., 16.}	32.9	0.151997	Reff	0.720881	0.687699	{0.678249, 0.699992}	4.603	ar	592.	543.47	{515., 577.}	8.52196
30	R0	{1.72795, 13.5635, 1.}	{1.51444, 16.0011, 5.}	{{1.51444, 16.0011, 5.}, {1.51444, 16.0011, 5.}}	{12.356, 17.9721, 400.}	inf	17.	20.5319	{17.2292, 23.737}	21.4067	0.0818369	newly	2.	8.22	{5., 11.}	311.	0.588436	Reff	0.60651	0.526221	{0.521306, 0.531204}	13.238	ar	668.	673.46	{659., 690.}	1.49102
31	R0	{1.51591, 18.1544, 3.}	{1.34505, 8.30068, 3.}	{{1.34668, 8.42466, 3.}, {1.34668, 8.42466, 3.}}	{11.2711, 54.2773, 1.33333}	inf	38.	26.8616	{17.1086, 33.8191}	29.9334	0.169208	newly	24.	20.48	{14., 29.}	22.3333	0.117512	Reff	0.858005	0.776261	{0.76548, 0.786596}	9.52717	ar	541.	492.08	{444., 540.}	9.6488
32	R0	{1.4282, 10.3534, 0.1}	{1.4396, 11.6213, 1.82}	{{1.44709, 12.3743, 2.}, {1.44709, 12.3743, 2.}}	{1.79409, 23.2789, 1738.}	inf	37.	54.7509	{46.4027, 65.8565}	58.1611	0.219125	newly	27.	31.03	{24., 38.}	22.7037	0.0941908	Reff	0.854064	0.835044	{0.821285, 0.846598}	2.7111	ar	521.	559.72	{506., 628.}	10.7025
33	R0	{1.56693, 11.3972, 0.1}	{1.68346, 10.6571, 2.62}	{{1.62191, 8.12461, 2.}, {1.77553, 15.0866, 4.}}	{7.43666, 29.1538, 2528.}	inf	21.	17.576	{6.87097, 30.9401}	47.6543	0.266525	newly	13.	9.29	{3., 15.}	34.2308	0.220767	Reff	0.518654	0.562265	{0.549541, 0.577349}	8.40858	ar	695.	683.85	{659., 716.}	3.31655
34	R0	{1.50675, 19.8823, 1.}	{1.47602, 3.95027, 0.02}	{{1.44762, 3.83841, 0.}, {1.53501, 4.06661, 0.}}	{2.45202, 80.1317, 100.}	inf	31.	6.73474	{2.6172, 11.565}	78.275	0.725767	newly	10.	9.73	{3., 18.}	46.1	0.241342	Reff	0.729267	0.750205	{0.737012, 0.769382}	2.88357	ar	587.	507.59	{489., 537.}	13.5281
35	R0	{1.72889, 7.76068, 0.1}	{1.77361, 14.2286, 4.}	{{1.77361, 14.2286, 4.}, {1.77361, 14.2286, 4.}}	{2.58644, 83.3424, 3900.}	inf	3.	36.9454	{32.8553, 40.9204}	1131.51	1.08856	newly	3.	16.13	{12., 20.}	437.667	0.722301	Reff	0.605112	0.560557	{0.553341, 0.567696}	7.36311	ar	650.	724.23	{707., 747.}	11.42
36	R0	{1.62644, 4.12303, 0.1}	{1.65456, 3.37285, 0.}	{{1.65456, 3.37285, 0.}, {1.65456, 3.37285, 0.}}	{1.72895, 18.1949, 100.}	inf	0.1	2.45687	{1.24442, 3.67858}	2356.87	1.35543	newly	0.1	3.48	{1., 7.}	3380.	1.45137	Reff	0.566001	0.571722	{0.569664, 0.573704}	1.01075	ar	652.	658.59	{653., 667.}	1.01074
37	R0	{1.70878, 15.8072, 1.}	{1.43027, 8.96989, 0.99}	{{1.42801, 9.02643, 1.}, {1.42801, 9.02643, 1.}}	{16.2985, 43.2544, 1.}	inf	40.	29.3728	{22.4757, 34.7236}	26.6173	0.14096	newly	11.	19.45	{13., 26.}	77.1818	0.236298	Reff	0.659589	0.567322	{0.558284, 0.575622}	14.0591	ar	674.	636.46	{614., 659.}	5.60534
38	R0	{1.58967, 6.80568, 0.1}	{1.54803, 5.0476, 3.29}	{{1.53501, 4.02511, 0.}, {1.55618, 5.30296, 4.}}	{2.62722, 25.8326, 3222.}	inf	5.	2.9727	{1.42526, 4.52957}	42.6539	0.276187	newly	3.	3.37	{1., 6.}	54.3333	0.278967	Reff	0.62474	0.61151	{0.606848, 0.617031}	2.19946	ar	610.	609.03	{604., 616.}	0.729508
39	R0	{1.344, 8.19498, 0.1}	{1.41037, 8.06114, 0.84}	{{1.38815, 6.71734, 0.}, {1.44476, 10.1243, 2.}}	{4.93817, 19.997, 858.}	inf	14.	10.7467	{3.84253, 15.8076}	31.158	0.199209	newly	14.	8.29	{3., 12.}	43.2143	0.308494	Reff	0.684096	0.722396	{0.708195, 0.737286}	5.59863	ar	551.	514.03	{484., 546.}	7.25045
40	R0	{1.61509, 7.90825, 0.1}	{1.50398, 3.80116, 0.13}	{{1.47488, 3.12918, 0.}, {1.53501, 4.02511, 0.}}	{7.12367, 51.9343, 210.}	inf	13.	7.78614	{2.69814, 13.4308}	43.3522	0.298302	newly	9.	10.64	{3., 19.}	51.7778	0.219564	Reff	0.7171	0.675601	{0.654702, 0.696598}	6.12419	ar	581.	566.68	{549., 588.}	3.51463
41	R0	{1.60017, 5.90605, 0.1}	{1.71337, 8.80249, 1.}	{{1.71337, 8.80249, 1.}, {1.71337, 8.80249, 1.}}	{7.07432, 49.042, 900.}	inf	6.	20.5251	{16.3613, 25.0822}	242.086	0.528322	newly	6.	13.49	{9., 17.}	124.833	0.335927	Reff	0.547548	0.561086	{0.55256, 0.567968}	2.55172	ar	661.	698.47	{682., 715.}	5.66868
42	R0	{1.64436, 15.1337, 1.}	{1.67657, 15.1267, 3.83}	{{1.39507, 4.06661, 0.}, {1.79789, 19.6311, 5.}}	{10.5522, 39.8677, 317.}	inf	40.	83.5839	{8.44366, 126.481}	158.179	0.5334	newly	15.	31.02	{7., 47.}	129.867	0.392893	Reff	0.779427	0.712706	{0.659774, 0.731593}	8.56028	ar	640.	678.46	{507., 775.}	18.6219
43	R0	{1.48917, 11.917, 0.1}	{1.37342, 6.31107, 1.65}	{{1.3618, 4.98002, 1.}, {1.40191, 6.77341, 2.}}	{7.77254, 47.0414, 1554.}	inf	13.	6.37941	{2.10601, 10.6907}	52.7225	0.391337	newly	9.	6.22	{1., 11.}	45.1111	0.328136	Reff	0.775858	0.724638	{0.715872, 0.738535}	6.62629	ar	551.	488.92	{470., 516.}	11.3938
44	R0	{1.65577, 7.89317, 1.}	{1.48036, 6.3449, 0.58}	{{1.44485, 4.06661, 0.}, {1.56097, 8.77207, 2.}}	{10.5937, 24.7373, 104.}	inf	7.	6.27807	{1.79718, 12.4336}	49.5853	0.271737	newly	5.	5.86	{2., 12.}	59.6	0.258275	Reff	0.738473	0.661175	{0.644351, 0.69024}	10.4673	ar	576.	564.62	{550., 586.}	2.90972
45	R0	{1.6909, 12.3275, 0.1}	{1.51324, 8.41526, 1.}	{{1.51295, 8.4085, 1.}, {1.51295, 8.4085, 1.}}	{10.5068, 31.7358, 900.}	inf	23.	10.0704	{5.20771, 15.337}	56.2157	0.394766	newly	8.	7.49	{3., 12.}	33.875	0.178568	Reff	0.654378	0.605192	{0.597031, 0.612239}	7.51646	ar	675.	613.9	{599., 628.}	9.05185
46	R0	{1.62351, 16.0698, 2.}	{1.33321, 8.63027, 2.71}	{{1.32705, 8.34284, 2.}, {1.34829, 9.33397, 3.}}	{17.8812, 46.2951, 35.5}	inf	51.	21.1451	{11.769, 31.0878}	58.539	0.419629	newly	13.	15.92	{7., 24.}	44.1538	0.173614	Reff	0.899425	0.778444	{0.760844, 0.798303}	13.4509	ar	591.	467.59	{428., 511.}	20.9729
47	R0	{1.52372, 4.38599, 0.1}	{1.52613, 4.01372, 0.}	{{1.47608, 4.02511, 0.}, {1.53501, 4.02511, 0.}}	{1.11614, 8.48761, 100.}	inf	0.1	2.30007	{1.29106, 3.50379}	2200.07	1.33132	newly	0.1	3.08	{1., 5.}	2980.	1.40358	Reff	0.582061	0.579473	{0.560264, 0.584375}	0.760234	ar	618.	624.52	{619., 634.}	1.05502
48	R0	{1.44306, 11.788, 1.}	{1.3875, 6.71902, 0.06}	{{1.38815, 6.71734, 0.}, {1.38815, 6.71734, 0.}}	{3.85027, 43.0012, 100.}	inf	22.	7.65702	{3.02237, 11.7255}	65.1954	0.514291	newly	12.	6.89	{3., 11.}	45.25	0.32254	Reff	0.722973	0.715034	{0.709561, 0.720394}	1.10833	ar	534.	501.58	{484., 527.}	6.29213
49	R0	{1.63624, 11.3893, 1.}	{1.61188, 8.7843, 3.}	{{1.61188, 8.7843, 3.}, {1.61188, 8.7843, 3.}}	{1.48888, 22.8724, 200.}	inf	22.	19.2007	{13.6945, 25.5377}	18.8284	0.0955225	newly	9.	12.83	{8., 17.}	48.5556	0.167162	Reff	0.623407	0.618638	{0.608184, 0.626428}	1.07054	ar	643.	642.08	{621., 665.}	2.14308
50	R0	{1.46113, 11.1322, 1.}	{1.27131, 3.63792, 0.}	{{1.22544, 3.07157, 0.}, {1.28596, 3.98513, 0.}}	{13.0119, 67.3207, 100.}	inf	28.	9.23948	{2.87116, 14.2221}	67.0018	0.549396	newly	17.	14.03	{3., 23.}	39.4706	0.24863	Reff	0.831383	0.747226	{0.721153, 0.761383}	10.218	ar	501.	438.4	{409., 474.}	12.7784
